# SP-D and CC-16 Pneumoproteins' Kinetics and Their Predictive Role During SARS-CoV-2 Infection

**DOI:** 10.3389/fmed.2021.761299

**Published:** 2022-02-08

**Authors:** Margherita Tiezzi, Sofia Morra, Jimmy Seminerio, Alain Van Muylem, Audrey Godefroid, Noémie Law-Weng-Sam, Anne Van Praet, Véronique Corbière, Carmen Orte Cano, Sina Karimi, Véronique Del Marmol, Benjamin Bondue, Mariam Benjelloun, Philomène Lavis, Françoise Mascart, Philippe van de Borne, Alessandra K. Cardozo

**Affiliations:** ^1^Department of Cardiology, Erasme University Hospital, Université Libre de Bruxelles, Brussels, Belgium; ^2^Inflammation and Cell Death Signalling Group, Experimental Gastroenterology Laboratory and Endotools-Medical Faculty, ULB, Brussels, Belgium; ^3^Institute for Translational Research in Cardiovascular and Respiratory Sciences, Université Libre de Bruxelles, Brussels, Belgium; ^4^Department of Respiratory Medicine, Erasme University Hospital, Université Libre de Bruxelles, Brussels, Belgium; ^5^Laboratory of Vaccinology and Mucosal Immunity, Université Libre de Bruxelles, Brussels, Belgium; ^6^Department of Dermatology, Erasme University Hospital, Université Libre de Bruxelles, Brussels, Belgium; ^7^Department of Internal Medicine, Erasme University Hospital, Université Libre de Bruxelles, Brussels, Belgium; ^8^Faculty of Medicine, Université Libre de Bruxelles, Brussels, Belgium; ^9^Department of Pathology, Erasme University Hospital, Université Libre de Bruxelles, Brussels, Belgium; ^10^Immunobiology Clinic, Erasme University Hospital, Université libre de Bruxelles, Brussels, Belgium

**Keywords:** COVID-19, club cell 16 protein, surfactant protein D, acute respiratory distress syndrome, biomarker, prognosis

## Abstract

**Background:**

Surfactant protein D (SP-D) and pulmonary club cell protein 16 (CC-16) are called “pneumoproteins” and are involved in host defense against oxidative stress, inflammation, and viral outbreak. This study aimed to determine the predictive value of these pneumoproteins on the incidence of acute respiratory distress syndrome (ARDS) or death in patients with coronavirus disease-2019 (COVID-19).

**Methods:**

This retrospective study included 87 patients admitted to an emergency department. Blood samples were collected on three time points (days 1, 5, and 14 from hospital admission). SP-D and CC-16 serum levels were determined, and univariate and multivariate analyses considering confounding variables (age, body mass index, tobacco use, dyspnea, hypertension, diabetes mellitus, neutrophil-to-lymphocyte ratio) were performed.

**Results:**

Based on the multivariate analysis, SP-D level on D1 was positively and slightly correlated with subsequent development of ARDS, independent of body mass index, dyspnea, and diabetes mellitus. CC-16 level on D1 was modestly and positively correlated with fatal outcome. A rise in SP-D between D1 and D5 and D1 and D14 had a strong negative association with incidence of ARDS. These associations were independent of tobacco use and neutrophil-to-lymphocyte ratio.

**Conclusions:**

Overall, our data reveal that increase in SP-D levels is a good prognostic factor for patients with COVID-19, and that initial CC-16 levels correlated with slightly higher risk of death. SP-D and CC-16 may prove useful to predict outcomes in patients with COVID-19.

## Introduction

A mature pulmonary epithelium is composed of several types of cells, such as specialized epithelial cells, goblet cells, basal cells, neuroendocrine cells, and club cells (also named Clara cells), to cite a few. These cells are differently distributed along the respiratory system and play a crucial role in fulfilling respiratory functions and maintaining the homeostasis of the respiratory system (1).

Club cells and non-ciliated bronchiolar cells synthetize the club cell 16 protein (CC-16), also known as uteroglobin, which is a 15.8-kDa homomeric lung protein encoded by the secretoglobin family 1A member 1 (SCGB1A1) gene (2–4). This protein is easily detectable in blood because of its transfer from the epithelial barrier to the blood stream, and it seems to be one of the most common proteins in the bronchoalveolar liquid (2, 4). Through its anti-inflammatory properties, CC-16 acts as part of the host's defense against several external stimuli, such as environmental agents, infections, oxidative stress, and inflammation (2, 4). CC-16 is, thus, considered as a useful biomarker for club cell dysfunction (5). Indeed, naive CC-16 knockout mice present altered lung function, enhanced remodeling characters, and increased risk of viral respiratory tract infection, witnessing its anti-inflammatory and anti-oxidative function (2, 4).

Serum CC-16 levels decrease in different chronic lung diseases, such as chronic pulmonary obstructive lung disease (COPD) (6, 7), asthma (8), and chronic exposure to different toxic agents (9–12), whereas higher CC-16 levels were noticed in patients with sarcoidosis (5, 13), pulmonary fibrosis (14), and acute exposure to smoke and toxic agents (15, 16). In smokers and COPD patients, CC-16 levels are linked to disease severity, showing a positive correlation with the levels of Forced Expiratory Volume (FEV1) in both COPD and asthma, and decreasing especially in patients with asthma COPD overlap syndrome. Moreover, serum CC-16 levels have been shown to correlate with lung infiltration in sarcoidosis (5, 13), and higher CC-16 levels have been found in acute lung injury (17), suggesting that it could be a marker of lung involvement and disease severity. Despite existing evidence in several acute and chronic lung injuries that globally suggests a protective role of CC-16 in lung parenchyma and lung function (4, 18), there is still lack of knowledge about the spectrum of its biological functions, and it is not yet clear whether CC-16 may represent a useful pathological marker for a lung injury with significant clinical implications.

Type II pneumocytes are specialized epithelial cells covering 5% of the alveolar surface; they are cuboidal in appearance and can differentiate into type I pneumocytes following a lung injury (1, 19). Besides their key role in cellular renewal, they secrete a surfactant into the surface of alveoli (20) containing lipids and proteins like surfactant protein D (SP-D). SP-D is a collagen-containing C-type lectin and member of the collectin family of proteins; it is involved in pulmonary innate immunity, displaying anti-inflammatory, and antioxidant properties. In particular, SP-D facilitates pathogen and apoptotic cell elimination, acts as a modulator of immune response through decrease of T cell proliferation and activation, reduces antigen presentation by macrophages (21–24), and globally assures anti-viral defenses through stimulation of virus agglutination and phagocytosis while limiting inflammatory cytokines and ROS production (22, 25). Structural alterations of SP-D can promote a proinflammatory state in mouse models of a lung injury (21): SP-D knockout mice present enhanced lung inflammation, macrophage activation, and ROS production, together with declined macrophage phagocytic ability (21, 22, 24).

Serum SP-D levels increase in chronic lung diseases such as COPD, asthma, idiopathic pulmonary fibrosis, sarcoidosis, and cystic fibrosis (26). Interestingly, the level of SP-D increase has recently been shown to correlate to ARDS etiology (bacterial, viral, and atypical), thus giving a rationale for molecular phenotyping and individualized treatments in this syndrome (27).

Acute respiratory distress syndrome (ARDS) is an acute respiratory system failure generated by non-cardiogenic lung edema (28, 29).

From a clinical point of view and according to Berlin Criteria, ARDS is defined as an acute onset of dyspnea associated with hypoxemia (arterial partial pressure of oxygen to fraction of inspired oxygen [PaO2/FIO2] < 300 mmHg) and bilateral infiltrates on chest radiograph, with no evidence of left atrial hypertension or cardiac failure (28–30). In particular, these radiological alterations represent ARDS anatomical features (28, 29), while ARDS immune-related lesions are represented by increased vascular and epithelial permeability secondary to overactive host inflammatory response to microbial aggression (28, 29). Viral and bacterial pneumonia are the most likely etiologies for ARDS (28, 29). Several studies propose CC-16 and SP-D as valuable diagnostic and prognostic markers of acute respiratory distress syndrome (ARDS) (26, 31, 32). ARDS is a frequent complication of coronavirus disease-2019 (COVID-19), occurring in up to 33% of hospitalized patients with SARS-CoV-2 infection (33). This infection, responsible for the actual COVID-19 pandemic, represents an ongoing and still evolving health problem without any approved treatment and controlled mainly by mass vaccination. The comprehension of this disease is still not complete, and new biomarkers might be needed to anticipate disease progression and predict outcomes in infected patients. Thanks to recent scientific evidence, it is already consolidated that there is high tropism of SARS-CoV-2 for the respiratory epithelium, where the virus binds the angiotensin converting enzyme-2 (ACE-2) receptor on lung cells representing the gateway into the respiratory epithelium (34). Recent studies already suggested a physiopathological mechanism for the fluctuation of pneumoprotein concentrations in COVID-19 (32) and partially discovered a correlation between circulating levels of pneumoprotein at baseline and ARDS onset (35). Investigations with strong statistical evidence for the influence of pneumoprotein kinetics on outcomes were lacking, so this research attempted to provide perspectives about the use of pneumoproteins as potential predictive biomarkers.

Acknowledging that CC-16 and SP-D are suggested as valuable biomarkers for ARDS, the first objective of this investigation is to investigate the behavior of these proteins according to the clinical evolution and severity of COVID-19.

### Study Design

This retrospective study included analysis of blood samples collected from patients who were consecutively admitted to a tertiary care center of Erasme Hospital (Brussels, Belgium) from April to December 2020 for confirmed/suspected SARS-CoV-2 infection. The study was approved by the local Ethical Committee ULB–Hôpital Erasme (aggregation number OMO21, study protocol P2020/238), and all procedures involving participants were performed in accordance to the Declaration of Helsinki.

**Figure 1:**
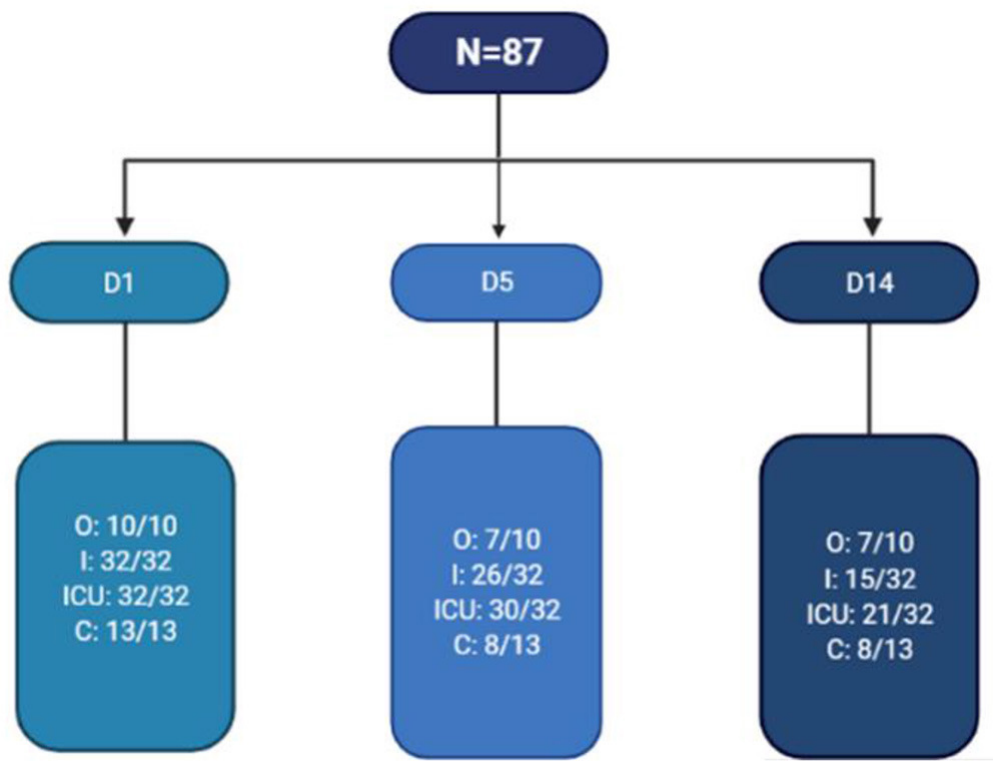
Flow chart of blood samples collected in this study. N, number of patients; D1, D5, and D14, days 1, 5, and 14 of hospitalization, respectively; O, outpatient; I, inpatient; ICU, intensive care unit; C, convalescent.

The blood samples were collected at three different timepoints and analyzed retrospectively: on D1, D5, and D14 (Figure 1). Of note, the blood samples were immediately treated (<2 h), and the sera were stored at −20°C in the Biobank (BB190012) at the Laboratory of Vaccinology and Immunology at the Université libre de Bruxelles prior to analysis.

We retrospectively selected patients with evidence of SARS-CoV-2 infection based on positive polymerase chain reaction (PCR) test and/or a suspected SARS-CoV-2 infection on lung CT scan. The selected participants were categorized retrospectively into four groups: the ambulatory group (outpatients, O), subjects who presented to the emergency room (ER) with mild symptoms, and discharged immediately after admission to the ER (*n* = 10); the hospitalized group (inpatients, I): patients hospitalized for moderate/severe COVID-19 who did not require intensive care unit stay during their hospitalization (*n* = 32); the intensive care unit group (ICU): patients hospitalized and admitted in the ICU on D1 for severe COVID-19 (*n* = 32); the convalescent group (C): subjects who presented to the ER with COVID-19-like symptoms lasting for at least more than 10 days and a negative PCR test (according to the hypothesis that the PCR test becomes usually negative within > 10 days) (*n* = 13) (36). Development of ARDS at any time during the course of the disease was evaluated retrospectively and in accordance to Berlin criteria, based on clinical, biological (arterial blood gas analysis), and radiological data.

### Clinical Features

Clinical characteristics were gathered from the local electronic medical record, and the following baseline characteristics were evaluated: age, gender, tobacco use, body mass index (BMI), past medical history, cardiovascular treatment, and symptoms on admission. Regarding past medical history, hypertension was defined as patients requiring anti-hypertensive therapy to maintain blood pressure under 140/90 mmHg; diabetes mellitus (DM) was defined as patients requiring oral antidiabetic drugs/insulin therapy and diagnosed prior to admission; asthma was defined as a reversible obstructive airway disease based on lung function tests prior to admission; heart failure (HF) was defined as patients suffering from cardiomyopathy and presenting with dyspnea and lower limb edema. Retained symptoms on admission were dyspnea (defined as a subjective complaint of respiratory system impairment), fever (defined as axillar temperature above 38°C), and cough.

### Routine Biological Analysis

A routine biological analysis was performed, and the following data were collected from the local electronic medical record: C-reactive protein (CRP), white blood cell count (WBC), polymorphonuclear leukocytes (PMN), lymphocytes, neutrophil-to-lymphocyte ratio (NLR), platelet (PLT), D-dimers, ferritin, creatinine, and glomerular filtration rate (GFR) calculated with the CKD-EPI formula.

### CC-16 and SP-D Analysis

The CC-16 protein was measured in serum using an ELISA kit (Cat No: RD191022200R; Biovendor^©^, Karasek, Czech Republic). Serum samples were diluted 1×, and the ELISA test was performed according to the instruction of the manufacturer. Each ELISA sample was run in duplicate. The SP-D protein was measured in serum using an ELISA kit (Cat No: RD194059101; Biovendor^©^, Karasek, Czech Republic). Serum samples were diluted 11×, and the ELISA test was performed according to instruction of the manufacturer. Each sample was run in duplicate. Of note, protein serum concentration below the lower detection limit was encoded as the value correspondent to the lower detection limit accordingly.

### Statistical Analysis

Descriptive statistics were generated for each patient group. Normality of distribution was tested using the Kolmogorov-Smirnov and Shapiro-Wilk tests. Parametric or non-parametric tests were performed according to data distribution. Differences among the four unpaired groups were tested by one-way analysis of variance or Kruskal-Wallis, and *post-hoc* pairwise comparisons of the groups were performed using the asymptotic significance (2-sided tests) with Bonferroni correction for multiple comparisons. Descriptive data and continuous variables are presented as mean ± standard deviation (SD) or median with a confidence interval of 95%. Categorical variables are presented as numbers with percentages.

To evaluate the temporal profiles of biological variables, a mixed linear model was used with groups and time points as fixed effects and variables measured on D1 as random effect (random-intercept model) variables. Prediction of ARDS and death was evaluated using a logistic regression model. Univariate analysis was first performed to assess significant explicative variables. Then, a multivariate analysis was performed using a backward stepwise procedure, the starting model including all significant variables (age, BMI, smoking, dyspnea, hypertension, DM, NLR at day 1 and day 5). All the statistical analyses were performed with IBM SPSS Statistics 27 and the R-software (version 4.0.3) for Windows. Significance was set at 0.05.

## Results

### Baseline Characteristics

Eighty-seven patients were enrolled in this research, and the baseline characteristics of the studied population are described in Table 1. Fifty-nine subjects were men (67.8%), 28 were women (32.2%), and their mean age was 54.5 ± 13.9 years. Current and former smokers represented 20.7% of the sample. The average BMI was 30.1 ± 6.7 kg/m^2^. Patients in the ICU group were older than patients in the other groups (ICU: 60.3 ± 11.4, inpatients: 57 ± 13.2; outpatients: 40.8 ± 12.8; convalescent: 44.5 ± 9.7). BMI was comparable among the groups. Routine biological analysis on day 1 as well as their kinetics over the observational period (D1, D5, and D14) are reported in Table 1.

**Table 1:** Baseline and clinical characteristics, anamnestic information, routine biomarkers and pneumoproteins on admission and outcome in our population.

**Variable**	**No (%) All (*n =* 87)**	**O (*n =* 10)**	**C (*n =* 13)**	**I (*n =* 32)**	**ICU (*n =* 32)**	**P_**all**_**	***P*-value ^**O vs. I**^**	***P*-value ^**O vs. ICU**^**	***P*-value ^**O vs. C**^**	***P*-value ^**I vs. ICU**^**	***P*-value ^**ICU vs. C**^**	***P*-value ^**I vs. C**^**
**Baseline characteristics**												
Age (years)	54.5 ± 13.9	40.8 ± 12.8	44.5 ± 9.7	57 ± 13.2	60.3 ± 11.4	<0.001	0.002	<0.001	1	1	<0.001	0.013
Men, *n* (%)	59 (67.8)	7 (70)	8 (61.5)	21 (65.6)	23 (71.9)	/	/	/	/	/	/	/
Women, *n* (%)	28 (32.2)	3 (30)	5 (38.5)	11 (34.4)	9 (28.1)	/	/	/	/	/	/	/
BMI (kg/m^2^)	30.1 ± 6.7	27.9 ± 5.9	29.6± 9.4	29.3 ± 6	32.4± 7.1	0.28	/	/	/	/	/	/
Tobacco use, *n* (%)	18 (20.7)	1 (10)	1 (7.7)	7 (21.9)	9 (28.1)	/	/	/	/	/	/	/
**Medical history**												
Hypertension	39 (44.8)	2 (20)	1 (7.7)	17 (53.1)	19 (59.4)	/	/	/	/	/	/	/
HF	11 (12.6)	0 (0)	0 (0)	5 (15.6)	6 (18.8)	/	/	/	/	/	/	/
DM	28 (32.2)	1 (10)	1 (7.7)	9 (28.1)	17 (53.1)	/	/	/	/	/	/	/
Asthma	12 (13.8)	1 (10)	1 (7.7)	6 (18.8)	4 (12.5)	/	/	/	/	/	/	/
**Medication on admission**												
ACEi	16 (18.4)	0 (0)	0 (0)	12 (37.5)	4 (12.5)	/	/	/	/	/	/	/
ARA II	2 (2.3)	0 (0)	0 (0)	1 (3.1)	1 (3.1)	/	/	/	/	/	/	/
BB	17 (19.5)	1 (10)	0 (0)	11 (34.4)	5 (15.6)	/	/	/	/	/	/	/
Ca blocker	17 (19.5)	1 (10)	0 (0)	9 (28.1)	7 (21.9)	/	/	/	/	/	/	/
**Clinical characteristics on admission**												
Dyspnea, *n* (%)	57 (65.5)	3 (30)	6 (46.2)	25 (78.1)	23 (71.9)	/	/	/	/	/	/	/
Fever, *n* (%)	38 (43.7)	7 (70)	1 (7.7)	19 (59.4)	13 (40.6)	/	/	/	/	/	/	/
Cough, *n* (%)	57 (65.5)	5 (50)	7 (53.8)	27 (84.4)	18 (56.3)	/	/	/	/	/	/	/
SatO_2_ (%)	94 [89.8; 98.4]	99.3 ± 0.71	98.9 [97.7; 99.6]	93 [90; 94.3]	82.4 ± 12.4	<0.001	<0.001	<0.001	1	0.084	<0.001	0.002
Sympton onset prior to admission (days)	7 [4; 12]	5.7 ± 3.8	25.1 ± 10.1	6.3 ± 4.1	7.5 ± 3.3	<0.001	1	1	<0.001	1	<0.001	<0.001
**Biological markers on admission**												
CRP (mg/L)	/	10.6 [2.3–49.1] (9)	1 [0.4–2.6] (13)	54.8 [15.6–192.4] (30)	126.1 [69.2–229.9] (32)	<0.001	<0.001	<0.001	<0.001	0.013	<0.001	<0.001
WBC (10∧3/mm^3^)	/	4.6 [3.7–5.8] (10)	7.1 [5.3–9.5] (12)	6.5 [4.2–10.1] (31)	9.2 [5.5–15.4] (32)	0.005	/	<0.001	/	0.008	/	/
PMN (absolute) (10∧3/mm^3^)	/	2.5 [1.7–3.8] (8)	4.2 [2.9–6.3] (10)	4.7 [2.7–8.4] (30)	8.2 [5.5–12.2] (31)	<0.001	0.006	<0.001	/	<0.001	<0.001	/
Lymphocytes (10∧3/mm^3^)	/	1.5 [1.1–2] (8)	2.3 [1.9–2.8] (11)	1.0 [0.6–1.6] (31)	0.9 [0.5–1.6] (31)	<0.001	/	/	/	/	<0.001	<0.001
Platelets (10∧3/mm^3^)	/	171.6 [103.8–283.9] (10)	232.3 [163.7–329.8] (12)	179.5 [125.4–257] (30)	254.2 [165.1–391.3] (32)	0.165	/	/	/	/	/	/
Ddimères (ng/ml)	/	/	/	879.1 [294.6–2623.8] (25)	1525.4 [423.7–5491.9] (29)	0.098	/	/	/	/	/	/
Ferritine (μg/L)	/	/	/	508.1 [234.2–1102.1] (27)	1021.7 [403.2–2589.2] (28)	0.004	/	/	/	0.004	/	/
Creatinine (mg/dl)	/	0.9 [0.7–1.2] (10)	0.8 [0.7–1] (13)	1 [0.6–1.5] (31)	1 [0.6–1.6] (32)	0.343	/	/	/	/	/	/
GFR* (ml/min/1.73m^2^)	/	90.4 [71.3–114.5] (10)	94.9 [80.9–111.4] (13)	71.8 [41.7–123.6] (30)	70.4 [43.7–113.5] (32)	0.24	/	/	/	/	/	/
**Pneumoproteins**												
CC-16 (ng/ml)	/	6.3 [3.7–10.6] (10)	7.9 [5.3–11.8] (13)	6.7 [3.9–11.5](30)	8.1 [3.8–17.4] (32)	0.653	/	/	/	/	/	/
SP-D (ng/ml)	/	73.2 [50.2–106.8] (10)	86.7 [46–163.4] (13)	144.9 [55–381.5] (32)	304.3 [134.4–688.8] (32)	<0,001	/	<0,001	/	0.002	<0,001	/
**Outcomes**												
Hospitalization lenght (days)	8 [2; 17.75]	1 [1; 1]	1[1; 1]	7 [6; 11]	20 [12; 33]	<0.001	0.003	<0.001	1	0.001	<0.001	0.001
ARDS	32 (36.8)	0	0	2 (6.3)	30 (93.8)	/	/	/	/	/	/	/
Death, *n* (%)	11 (12.6)	0 (0)	0 (0)	0 (0)	11 (34.4)	/	/	/	/	/	/	/

*N, number of patients; O, outpatient; C, convalescent; ICU, intensive care unit; I, inpatients; BMI, body mass index; HF, heart failure; ACEi, angiotensin converting enzyme inhibitor; ARA II, angiotensin receptor antagonist; BB, beta blocker; CRP, C reactive protein; WBC, white blood cell count; PMN, polymorphonuclear; GFR, glomerular filtration rate (following CKD-EPI formula); CC-16, club cell secretory protein 16; SP-D, surfactant protein-D; ARDS, acute respiratory distress syndrome*.

### Clinical Characteristics on Admission

Dyspnea and cough occurred both in 65.5% of the subjects, while fever manifested in 43.7% of the subjects. Median arterial oxygen saturation (SatO_2_) was 94% [89.8; 98.4]: ICU and hospitalized patients disclosed lower values than the outpatients and convalescent patients (*p*_*all*_<*0.0*01) (Table 1). The patients presented first signs of infection in an average of 7 days [4, 12] before seeking medical attention, so D1, D5, and D14 correspond to the 7th, 12th, and 21st day from symptom onset, respectively.

### Medical History, Cardiovascular Therapy, and Outcomes of the Studied Population

Concerning their past medical history, the most prevalent comorbidities found in the study population are hypertension (44.8%), DM (32.2%), asthma (13.8%), and HF (12.6%) (Table 1). According to group allocation, hypertension was found in 53.1 and 59.4%; DM was found in 28.1 and 53.1%; asthma in 18.8 and 12.5%, and HF in 15.6 and 18.8% of hospitalized and ICU patients, respectively.

Regarding cardiovascular therapy, 18.4% of the patients were under angiotensin converting enzyme inhibitors (ACEi) treatment, of which 12 and 4 patients belonged to the hospitalized and ICU groups, respectively;19.5% of the patients were under Ca^2+^channel blockers, of which 1 was in the outpatient group, 9 were in the hospitalized, and 7 were in the ICU groups;19.5% of the patients were under beta blockers, of which 1 was in the outpatient, 11 were in the hospitalized, and 5 were in the ICU groups (Table 1).

In the whole studied sample, ARDS occurred in 36,8% of the patients, of whom 2 and 30 subjects belonged to the hospitalized and ICU groups, respectively; 12.6% of the patients had a fatal outcome; all of them belonged to the ICU group (Table 1).

### CC-16 Analysis

No significant differences in serum CC-16 levels on D1 are found among the groups (Table 1). Regarding the CC-16 kinetics over the observational period, in the hospitalized group, the serum levels of CC-16 increased on D14 (9.7 [6.2–15.2] ng/ml) compared to those on D1 (6.7 [3.9–11.5] ng/ml; *p* = 0.001), while in the ICU group, these increased on D5 (11.4 [4.8–26.9] ng/ml; *p* < 0.001) and D14 (17.1 [6.8–43.5] ng/ml; *p* < 0.001) compared to those on D1 (8.1 [3–17.4] ng/ml) (Table 2). Regarding ARDS prediction, the univariate analysis revealed that an upward trend of CC-16 levels between D1 and D5 was associated with a 4-fold increased risk of developing ARDS (OR: 3.83 [1.38–11.61]; *p* = 0.013). Changes in CC-16 levels between D1 and D14 were not associated to risk of ARDS (Table 3). However, the association between CC-16 circulating levels and ARDS onset was lost at the multivariate analysis (Table 5). Regarding death prediction, in the univariate analysis, CC-16 levels on D1 were associated with higher risk of death: for every unit increase in CC-16, the risk of death was multiplied by 1.13 (OR: 1.13 [1.04–1.25]; *p* = 0.006; Table 4). This association was also confirmed in the multivariate analysis after correction of the confounding factors, with an OR of 1.17 [1.04–1.35] (*p* = 0.011) (Table 6).

**Table 2:** Temporal profiles of CC-16 and SP-D according to the group.

		**D1**	**D5**	**D14**
CC-16
	O	6.3 [3.7–10.6]	6 [4–9]	7.8 [5.4–11.3]
	I	6.7 [3.9–11.5]	7.5 [4.6–12.5]	9.7 [6.2–15.2]^†^
	ICU	8.1 [3.8–17.4]	11.4 [4.8–26.9]^‡^	17.1 [6.8–43.5]^‡^
	C	7.9 [5.3–11.8]	8.7 [6.5–11.6]	9.7 [6.9–13.7]
	*P*-value	0.653		
SP-D
	O	73.2 [50.2–106.8]	76 [55.7–103.7]	66.8 [48.8–91.4]
	I	144.9 [55–381.5]	296.3 [162–541.8]^‡^	242.8 [105.1–560.8]^†^
	ICU	304.3 [134.4–688.8]	271.7 [94.4–781.9]	219.8 [109.2–442.4]
	C	86.7 [46–163.4]	101.5 [49.4–208.6]	
	*P*-value	<0.001		

A mixed linear model was used, with groups and time points as fixed effects and variables measured at D1 as random effects (random-intercept model). D1, D5 and D14, day 1, day 5 and day 14 of hospitalization respectively; O, outpatient; C, convalescent; ICU, intensive care unit; I, inpatients. *indicates p-value < 0.05, **indicates p-value < 0.01.

**Table 3:** Risk for ARDS: Univariate analysis.

	**OR**	**95% CI**	***P*-value**
CC-16 (D1)	1.063	0.993–1.15	0.092
SP-D (D1)	1.006	1.003–1.009	<0.001
SP-D/CC-16 (D1)	1.031	1.015–1.052	<0.001
CC-16 kinetics (D1–D5)	3.83	1.38–11.61	0.013
CC-16 kinetics (D1–D14)	1.53	0.28–12.3	0.617
SP-D kinetics (D1–D5)	0.106	0.03–0.326	<0.001
SP-D kinetics (D1–D14)	0.181	0.051–0.59	0.006
SP-D/CC-16 kinetics (D1–D5)	0.240	0.083–0.654	0.006
PMN/lymphocytes D1	1.20	1.10–1.35	<0.001
Age	1.05	1.01–1.09	0.010
Sex	1.35	0.53–3.60	0.537
BMI	1.10	1.02–1.21	0.018
Smoking	6.11	1.93–20.62	0.003
Dyspnea	14.21	2.67–263.46	0.012
Fever	1.63	0.59–4.70	0.355
Cough	1.15	0.395–3.68	0.799
HTA	2.91	1.15–7.72	0.027
DM	4.09	1.59–10.99	0.004

*OR, odds ratio; CI, confidence interval; D1, D5 and D14, day 1, day 5 and day 14 of hospitalization respectively; CC-16, club cell secretory protein 16; SP-D, surfactant protein D; PMN/lymphocytes, polymorphonuclear to lymphocytes ratio; BMI, body mass index; HTA, arterial hypertension; DM, diabetes mellitus*.

**Table 4:** Risk for death: Univariate analysis.

	**OR**	**95% CI**	***P*-value**
CC-16 (D1)	1.13	1.04–1.25	0.006
SP-D (D1)	1,000	0.999–1.001	0.163
SP-D/CC-16 (D1)	0.999	0.998–1.001	0.756
CC-16 kinetics (D1–D5)	3.15	0.71–22.08	0.168
CC-16 kinetics (D1–D14)	/	/	> 0.999^*^
SP-D kinetics (D1–D5)	1.06	0.26–5.3	0.942
SP-D kinetics (D1–D14)	0.92	0.18–5.16	0.992
SP-D/CC-16 kinetics (D1–D5)	1.14	0.29–4.85	0.854
PMN/lymphocytes D1	1.12	1.05–1.22	0.001
Age	1.04	0.99–1.1	0.129
Sex	2.25	0.53–15.49	0.322
BMI	1.15	1.07–1.34	0.044
Smoking	/	/	0.014^*^
Dyspnea	1.58	0.21–31.97	0.688
Fever	1.79	0.16–35.53	0.641
Cough	/	/	0.315
HTA	0.89	0.21–3.62	0.871
DM	2.26	0.58–8.88	0.230

*OR, odds ratio; CI, confidence interval; D1, D5 and D14, day 1, day 5 and day 14 of hospitalization respectively; CC-16, club cell secretory protein 16; SP-D, surfactant protein D; PMN/lymphocytes, polymorphonuclear to lymphocytes ratio; BMI, body mass index; HTA, arterial hypertension; DM, diabetes mellitus*.

* * = Fisher test*.

**Table 5:** Risk for ARDS: Multivariate analysis.

	**OR**	**95% CI**	***P*-value**
CC-16 (D1)	/	/	/
SP-D (D1)	1.007	1.003–1.014	0.006
SP-D/CC-16 (D1)			
CC-16 kinetics (D1–D5)	/	/	/
CC-16 kinetics (D1–D14)			
SP-D kinetics (D1–D5)	0.023	0.003–0.12	<0.001
SP-D kinetics (D1–D14)	0.041	0.002–0.29	0.007
SP-D/CC-16 kinetics (D1–D5)	0.17	0.037–0.66	0.012

*Confounding factors taken into account in the model are listed hereafter. SP-D, D1, SP-D, BMI, dyspnea, DM; SP-D D1 to D5 and D1 to D14, smoking; OR, odds ratio; CI, confidence interval; D1, D5 and D14, day 1, day 5 and day 14 of hospitalization respectively; CC-16, club cell secretory protein 16; SP-D, surfactant protein D; BMI, body mass index; DM, diabetes mellitus*.

**Table 6:** Risk for death: Multivariate analysis.

	**OR**	**95% CI**	***P-value***
CC-16 (D1)	1.17	1.04–1.35	*0.011*

*Confounding variable taken into account for CC-16 at D1 was BMI. OR, odds ratio; CI, confidence interval; D1, D5 and D14, day 1, day 5 and day 14 of hospitalization respectively; BMI, body mass index*.

### SP-D Analysis

On day 1 of hospital admission, SP-D levels were higher in the ICU patients (304.3 [134.4–688.8] ng/ml) compared to those of the other groups: outpatient group (73.2 [50.2–106.8] ng/ml; *p* < 0.001); hospitalized group (144.9 [55–381.5] ng/ml; *p* = 0.002); convalescent group (86.7 [46–163.4] ng/ml; *p* < 0.001) (Table 1). Regarding the SP-D kinetics over the observational period, in the hospitalized group, SP-D increased on D5 (296.3 [162–541.8] ng/ml; *p* < 0.001) and D14 (242.8 [105.1–560.8] ng/ml; *p* = 0.007) compared to that on D1 (144.9 [55–381.5] ng/ml) (Table 2).

Regarding ARDS prediction, the univariate analysis showed that on D1, SP-D serum levels were positively associated to ARDS onset (OR: 1.006 [1.003–1.009]; *p* < 0.001) (Table 3). Conversely, the rise in circulating SP-D levels between D1 and D5 and between D1 and D14 was, respectively, associated with reduced risk of ARDS onset by one-tenth and one-fifth (D1 to D5 OR was 0.106 [0.03–0.326]; *p* < 0.001; D1 to D14 OR was 0.181 [0.051–0.59]; *p* =0.006) (Table 3). These associations were confirmed in the multivariate analysis after correction for the confounding factors. Indeed, on D1, for every increase of one unit of SP-D, the risk of developing ARDS was multiplied by 1.007 (OR: 1.007 [1.003–1.014]; *p* = 0.006) (Table 5). Regarding the SP-D kinetic in the multivariate analysis, each unit rise in SP-D level between D1 and D5 was associated with a 40-fold lower risk of developing ARDS (OR:0.023 [0.003–0.12]; *p* < 0.001). The same trend was observed for the SP-D kinetic between D1 and D14: each extra unit of SP-D was associated with a 20-fold lower risk of developing ARDS (OR:0.041 [0.002–0.29]; *p* = 0.007) (Table 5). A further multivariate analysis evaluating the independence of SP-D kinetic for predicting ARDS onset was performed thereafter. Considering increasing SP-D levels and the neutrophil-to-lymphocyte ratio on D5 as confounding variables, increasing SP-D levels between D1 and D5 was associated with a 75-fold decreased risk of developing ARDS (OR:0.013 [0.0006–0.1]; *p* < 0.001). In a consistent manner, increasing circulating levels in the kinetic of SP-D between D1 and D14 was associated with a 12-fold lower risk of ARDS onset (0.077 [0.004–0.24]; *p* = 0.077), with the neutrophil-to-lymphocyte ratio on D1 and increase in SP-D circulating levels as confounding variables. Regarding death prediction, SP-D levels showed no association to this outcome, both in the univariate and multivariate analyses.

### SP-D/CC-16 Ratio

As SP-D and CC-16 levels seem to reflect different mechanisms of lung damage, we decided to analyze the ratio between these two pneumoproteins (37, 38).

In the univariate analysis, on D1, the high SP-D/CC-16 ratio was mildly and positively associated to the development of ARDS (OR: 1.031 [1.015–1.052]; *p* < 0.001). Conversely, the rise in SP-D/CC-16 ratio between D1 and D5 was associated with a 25-fold lower risk of developing ARDS (OR:0.24 [0.083–0.654]*; p* = 0.006) (Table 3). The multivariate analysis confirmed this result, showing that an increase in SP-D/CC-16 ratio between D1 and D5 was associated with a 20-fold lower risk of developing ARDS (OR:0.17 [0.037–0.66]; *p* = 0.012) (Table 6). Regarding death prediction, SP-D/CC-16 ratio was not associated to risk of death, neither in the univariate nor the multivariate analysis.

### Confounding Factor Variables

Regarding the possible effect of confounding factors on ARDS onset, the univariate analysis showed that age, BMI, smoking, dyspnea, hypertension, and DM were differently associated with higher risk of developing ARDS (Table 3). Regarding the possible effect of confounding factors on the risk of death, the univariate analysis revealed that a patient with a BMI 5-kg/m^2^ higher than that of another patient without any differences in the other confounding variables had an increased risk of death (OR: 1.15 [1.07–1.34]; *p* = 0.044) (Table 4). Additionally, according to the univariate analysis (*p* = 0.014) (Table 4) former and current smokers had a higher risk of death compared to patients who never smoked.

Based on univariate analysis, higher neutrophil-to-lymphocyte ratio on D1 witnessed a greater risk of ARDS onset and death. More precisely, each extra unit of neutrophil-to-lymphocyte ratio multiplied by 1.2 the risk of ARDS onset (OR: 1.2 [1.1–1.35]; *p* < 0.001) and by 1.12 the risk of death (OR: 1.12 [1.05–1.22]; *p* = 0.001). Similarly, the increased neutrophil-to-lymphocyte ratio on D5 was associated with a 1.2-fold increased risk of ARDS onset (1.2 [1.08–1.38]; *p* = 0.005), but the neutrophil-to-lymphocyte ratio on day 5 was not associated with death occurrence.

## Discussion

To our knowledge, this is one of the first investigations to demonstrate that SP-D and CC-16 kinetics during the time course of COVID-19 may have a predictive value for disease outcome. Our main results are summarized as follows: (1) higher baseline levels of SP-D in serum on the day of hospital admission is an independent but weak risk factor for developing ARDS; (2) on the other hand, the kinetics of circulating SP-D during SARS-CoV-2 infection is an independent protective element from developing ARDS, reducing the risk of ARDS by a factor of 50 when it increases from D1 to D5 of analysis (7–12 days after first symptoms). Further multivariate analyses showed the independence of SP-D kinetic for the prediction of ARDS onset, considering neutrophil-to-lymphocyte ratio as another confounding variable; (3) a rise in SP-D/CC-16 ratio from days 7 to 12 since symptom onset (D1 to D5 in the analysis) lessens the risk of ARDS by about one-fifth; (4) elevated levels of CC-16 on the day of admission are associated with a greater risk of fatal outcome. Thus, in patients with COVID-19 who did not experience ARDS, CC-16 increased modestly (45%) and late (21 days after symptoms onset, D14 in the analysis), while in patients with ARDS, CC-16 increased by 110% at this timepoint. In the case of mild COVID-19 or post infection, serum CC-16 levels are stable throughout the observed period.

As mentioned above we observed that higher baseline levels of SP-D result in a slightly enhanced risk of ARDS onset, while an increase in SP-D levels in the course of the disease lessens the risk of ARDS in patients with COVD-19. In agreement with our results, two recently published studies also correlated increased levels of SP-D with the severity of COVID-19 (39, 40). The first study observed significantly increased levels of SP-D on admission in infected patients, especially the ones developing ARDS and macrophages activation syndrome (MAS), followed by a slight decrease on day 5 after hospitalization. The second one observed in severe cases a significant increase in SP-D levels on admission, with positive correlation with inflammation markers (C-reactive protein, IL-6), but could not prove the protective role of this protein in COVID-19 (39, 40). The reasons for the differences between these studies and the present one could be related to multiple factors: a younger study population (mean age 49 years for Kerget and Tong's studies; mean age 59 years for our population); ethnical differences (Turkish and Chinese population, respectively); different treatments received during in-hospital stay: some patients from Kerget et al. were treated for suspected MAS with tocilizumab (an anti-IL-6 treatment), with a concomitant decrease in SP-D levels, which was not the case in our cohort. These trials also display differences in endpoints, with SP-D being dosed essentially on admission and recovery for Tong's study and at 2 in-hospital endpoints for Kerget, while more specific kinetics during the active phase of infection was realized in our project. Moreover, in our study, SP-D kinetics was analyzed in light of CC-16 alterations as well, which could contribute to improve the understanding of the role of pneumoproteins as well as lung injury by considering both permeability and cellular alterations.

Our observation indicating that increase levels of SP-D in the course of the disease is a favorable prognostic factor for patients with COVD-19 is in agreement with a protective “damage-limiting” role of SP-D (2, 35, 41–43). Previous investigations on SARS-CoV infection demonstrated that SP-D has binding sites for the spike glycoprotein of SARS-CoV with a positive correlation between anti-SARS-CoV specific antibodies and SP-D levels (44). Thus, it is possible that one of the beneficial effects observed for SP-D is due to its binding to the spike glycoprotein of SARS-CoV-2, facilitating viral clearance (22). SP-D serum levels were also shown to be increased in influenza A virus, respiratory syncytial virus (RSV), and human immunodeficiency virus (HIV) infections, enhancing their clearance from mucosal points of entry and modulating inflammatory response (22). Moreover, a recombinant fragment of human (rfh) SP-D has been proved to downregulate inflammation during influenza A infection *in vitro* (45, 46) and has recently been shown to neutralize SARS-CoV-2 *in vitro* (47). We could, thus, speculate that SP-D increases in the acute phase in parallel with the extent of lung injury, and then acts mostly as an immunomodulator limiting excessive parenchymal and airway damage later in the time course of the disease.

In response to ARDS, increasing levels of SP-D may also represent proliferation of type II pneumocytes and reflect the regeneration capacity of the lung parenchyma (48). Type II pneumocytes also ensure the maintenance of alveolar barrier integrity and host defense against aggression. Thus, we propose that rising SP-D levels early over the time course of SARS-CoV-2 infection may reflect the residual capacity of type II pneumocytes to secrete SP-D, thus ensuring regeneration of the lung parenchyma. More precisely, early increase in circulating levels of SP-D may reflect an adequate response of the lungs to viral aggression, reflecting the regeneration of surfactant-secreting cells. Nevertheless, higher levels of SP-D on admission may be explained by the translocation of SP-D from the alveolar epithelium to blood circulation because of loss of the alveolo-capillary barrier integrity and, thus, explain the very mild predictive value for ARDS onset. Recent research on COVID-19 pathogenesis proposed that loss of alveolar cell integrity, loss of gas exchange capacity, and development of ARDS may be also consequences of the downregulation of transcription factors implicated in SP-D synthesis (43).

As explained above, CC-16 also displays a protective role toward lung parenchyma, mirroring the extent of cell loss during lung damage and being tightly linked to lung function (2, 35, 41–43). This investigation recognizes the circulating levels of CC-16 on day ± 7 after symptom onset as a mild independent factor of death, and provides a new perspective regarding the predictive value of CC-16 for survival outcome in patients with COVID-19. However, conversely to SP-D, CC-16 kinetics was not found to have any predictive impact on ARDS onset or death. A previous study including several etiologies of ARDS has demonstrated that CC-16 at baseline was correlated with ARDS onset based on univariate analysis but lost its predictive value on multivariate analysis (48). This is in line with our results, where we found that early rise of circulating levels of CC-16 is associated to a 4-fold risk of developing ARDS in univariate analysis, but not in multivariate analysis. Lin et al. also reported a predictive value of CC-16 level at baseline on ARDS severity: the higher the circulating levels of CC-16, the more severe the ARDS (32).

Autoptic studies demonstrated that COVID-19 progression is associated with excessive mucin production predominantly in distal airways (43). Mucin genes seem to be upregulated in club cells and the excessive mucus increases the viscosity of lung secretions and impairs mucociliary clearance. This enhancement of mucus secretion observed in COVID-19 is probably due to innate immunity and may favor ARDS onset (43). We suggest that while increased circulating levels of SP-D may reflect renewal of the lung parenchyma, high circulating levels of CC-16 may reflect the expansion of lung injury from alveolar tissue to airways. The damage on club cells in this stage of the disease is probably so important that the regenerative properties of these cells may be overcome.

Interestingly, in our study, the levels of SP-D and CC-16 displayed different temporal patterns according to the subjects studied. Thus, in patients requiring intensive care, SP-D serum level did not change over the observed period, while the CC-16 serum level progressively increased (Table 2). Conversely, in hospitalized patients without ARDS, SP-D levels rose early during SARS-CoV-2 infection on day 5 (± 12 days after symptoms onset) and lessened afterward, while the CC-16 rose lately on day 14 (± 21 days after symptoms onset). CC-16 serum levels could be affected both by club cells damage/loss and increased/decreased epithelial barrier permeability, being decreased in the case of cellular loss and increased in the case of epithelial cell stress or in the case of higher permeability of the air-blood barrier. Serum levels of SP-D, on the other hand, increased secondary to higher epithelial barrier permeability and were less affected by epithelial cell loss (10). For this reason, the serum SP-D/CC-16 ratio has been introduced to integrate both club cell damage (reflected by CC-16 levels) and changes in epithelial barrier permeability (witnessed mainly by SP-D levels). Thus, SP-D/CC-16 ratio is a useful tool to understand the mechanisms of lung damage: it is expected to decrease when the stressor causes acute cellular stress (higher CC-16 levels) (37) with less effects on epithelial permeability (low SP-D levels), and to increase when the damage provokes club cell loss (low CC-16 levels) and/or when it mostly affects permeability (higher SP-D levels) (38).

In our study, we found a double-sided behavior of this ratio: high SP-D/CC-16 ratio being positively correlated to the development of ARDS in the acute phase (D1), while rise of this ratio between D1 and D5 appeared to be protective. Our interpretation of these results is that in the acute phase, SP-D/CC-16 ratio increases as a result of lung injury with increased permeability and cell loss, thus representing a risk factor for developing ARDS (the more extended the damage, the more likely to develop severe lung injury). On the other hand, later in the time course of the disease, SP-D/CC-16 increase appears to be protective, possibly showing that in this case a rise in serum SP-D is not just a reflex of hyperpermeability but also of hyperproduction of SP-D secondary to lung damage (the lungs respond to the injury *via* SP-D upregulation to modulate inflammatory mechanisms). This interpretation is supported by evidence of high SP-D levels in ARDS acute phase (as a result of a rupture of air-blood barrier) (49) and hyperproliferation of type II pneumocytes found in the proliferative phase of ARDS (occurring around 7 days after injury), which could lead to subsequent SP-D hyperproduction (50).

However, to this date, there has been lack of scientific evidence about the use of SP-D/CC-16 ratio to predict outcomes or characterize respiratory disease; therefore, further studies especially focusing on ARDS prediction and ARDS phenotyping are necessary.

### Limitations

This study encounters several limitations. This is a retrospective study that includes a small number of patients who presented to the ER of a tertiary care center for suspicion of SARS-CoV-2 infection, secondary confirmed based on PCR test and/or suspected SARS-CoV-2 infection on lung CT scan. Thus, the population included in this investigation may not be representative, and the results may not be reproductible in other research centers. However, baseline, clinical characteristics, and medical history were comparable to other large-scale studies (51–53). No bronchoalveolar liquid samples were collected from the patients during this research; thus, there is lack of comparison between circulating levels of SP-D and CC-16 and bronchoalveolar liquid concentration. Confounding variables were not distributed randomly in the different subgroups. However, independent predictive factors were tested by multivariate analysis, which lessen the impact of confounding variables. The recruitment period was short, from April to December 2020. Regarding CC-16, the circulating levels may be affected by renal clearance, with low renal clearance associated with higher circulating levels of CC-16 (54). However, in our cohort, only 6% of the patients suffered from renal function impairment, thus lowering the impact of this confounding variable. Despite the existence of stratification for ARDS severity according to the Berlin criteria (30), the authors were not able to classify the patients as “mild,” “moderate,” or “severe,” ARDS, as the subjects were enrolled retrospectively, and their respiratory conditions were already stabilized under mechanical assistance prior to the enrollment, and biological data of arterial blood gas exchanges were often missing. However, we believe that these limitations do not preclude our conclusions.

## Conclusions

To conclude, circulating levels of SP-D and CC-16 and their temporal profiles over the course of COVID-19 may be useful as biomarkers for ARDS and death prediction. While increased SP-D levels appear to be predictive of positive outcome, the opposite was observed regarding CC-16 levels. Further longitudinal multicentric studies that include a higher number of subjects with different ethnicities are needed to confirm the role of SP-D and CC-16 in the time course of COVD-19.

## Data Availability Statement

The raw data supporting the conclusions of this article will be made available by the authors, without undue reservation.

## Ethics Statement

The studies involving human participants were reviewed and approved by Erasme Hospital Ethics Committee (reference number (P2020/238). The patients/participants provided their written informed consent to participate in this study.

## Author Contributions

The protocol was designed by SM, PB, VC, and AC. The biological measurements of SP-D and CC-16 were carried out by SM, AG, and NL-W-S with valuable support from VC. Clinical data collection was performed by SK, CC, and JS. The bio banking of blood samples was realized by SM, AG, and AP. Database management was performed by SM, JS, MB, CC, and PL. Analysis and interpretation of the data were performed by SM, JS, NL-W-S, VC, FM, and AC. Statistical analysis were performed by AM, with contribution from JS. The manuscript was written by SM, AC, MT, and JS. All authors critically revised the manuscript, contributed to the article, and approved the submitted version.

## Funding

This research was supported by Association Jaumotte (VM, CC, SK, and VC), Fonds Erasme (SM, AC, PB, and NL-W-S) and Special COVID-19 fund U.L.B (FM, BB).

## Conflict of Interest

The authors declare that the research was conducted in the absence of any commercial or financial relationships that could be construed as a potential conflict of interest.

## Publisher's Note

All claims expressed in this article are solely those of the authors and do not necessarily represent those of their affiliated organizations, or those of the publisher, the editors and the reviewers. Any product that may be evaluated in this article, or claim that may be made by its manufacturer, is not guaranteed or endorsed by the publisher.

## Abbreviations

ACE-2: angiotensin converting enzyme - 2
ACEi: angiotensin converting enzyme inhibitors
ARDS: acute respiratory distress syndrome
BMI: body mass index
CC-16: club cell secretory protein 16
COPD: chronic pulmonary obstructive lung disease
COVID-19: coronavirus disease-2019
CRP: C-reactive protein
CT: computed tomography
ELISA: enzyme-linked immunosorbent assay
ER: emergency room
FEV1: forced expiratory volume in the first second
GFR: glomerular filtration rate
PaO2/FIO2: arterial partial pressure of oxygen to fraction of inspired oxygen
NLR: neutrophil-to-lymphocyte ratio
PCR: polymerase chain reaction
PEEP: positive end expiratory pressure
PLT: platelet
PMN: polymorphonuclear leukocytes
SaO2: arterial saturation level of oxygen
SP-D: surfactant protein D
WBC: white blood count.
